# Electromechanical Coupling and Piezoelectric Behaviour of (PDMS)–Graphene Elastomer Nanocomposites

**DOI:** 10.3390/polym18050623

**Published:** 2026-03-02

**Authors:** Murat Çelik, Miguel A. Lopez-Manchado, Raquel Verdejo

**Affiliations:** Institute of Polymer Science and Technology (ICTP), CSIC, Juan de la Cierva 3, 28006 Madrid, Spain; lmanchado@ictp.csic.es

**Keywords:** PDMS–graphene nanocomposites, electromechanical coupling, hyperelastic modelling, finite element simulation

## Abstract

Elastomer-based nanocomposites combining polymer flexibility with conductive nanofillers provide lightweight, stretchable systems with tunable electromechanical properties for wearable electronics, soft robotics, and self-powered sensors. However, predicting their nonlinear response remains challenging because the observed piezoelectric-like response arises from strain-dependent interfacial polarization and evolving piezoresistive conduction pathways within heterogeneous microstructures. We introduce a continuum electro-hyperelastic framework combining the Mooney–Rivlin model for large-strain elasticity with a Helmholtz free-energy approach for electrostatic coupling. Analytical expressions for stress, electric displacement, and apparent piezoelectric coefficients are derived and implemented in finite element simulations. The model accurately reproduces the experimental mechanical, dielectric, and electromechanical behaviour of polydimethylsiloxane (PDMS) nanocomposites with 0.1–1 wt% graphene. These show increased stiffness, relative permittivity (from 3.4 to 4.0, ≈18%), and quasi-static d_33_ coefficients (from −5.6 to −10.0 pC N^−1^, ≈80% enhancement). Analytical and finite element method (FEM) results show consistent trends across the full deformation range, with Maxwell stress agreement within 10% at lower deformation levels, while deviations of 33–40% for coupled electromechanical quantities at an axial displacement u_z_ = ~−1 mm (~16.7% compressive strain) are attributable to three-dimensional shear effects absent from the uniaxial analytical assumption. Simulations reveal that graphene boosts Maxwell stress, yielding a four-fold increase at lower stretch ratios. This reframes PDMS–graphene composites as electro-hyperelastic materials, offering a predictive, extensible framework. It highlights apparent piezoelectricity as an emergent, tunable effect from charge redistribution in a compliant hyperelastic matrix—guiding the design of next-generation flexible devices leveraging field-induced coupling over intrinsic polarization.

## 1. Introduction

The development of flexible piezoelectric composites has emerged as a promising strategy for next-generation energy harvesting, sensing, and actuation technologies. Unlike conventional ceramic piezoelectrics such as lead zirconate titanate (PZT), elastomeric polymer composites provide lightweight, deformable, and easily processable alternatives, enabling seamless integration into wearable and soft robotics. For their successful implementation, however, it is essential to accurately predict and optimise the electromechanical response under complex loading conditions, which requires robust theoretical and numerical modelling frameworks.

Polydimethylsiloxane (PDMS) is among the most widely used elastomeric matrices due to its high compliance, chemical inertness, optical transparency, and biocompatibility, with well-documented performance in microfluidics and soft devices [1,2,3]. Reinforcement with graphene fillers significantly enhances the mechanical stiffness, dielectric permittivity, and electrical conductivity of PDMS composites [4,5]. These improvements primarily arise from interfacial polarization effects and percolation phenomena, making PDMS–graphene elastomer composites (PGECs) attractive candidates for soft energy harvesters, sensors, and actuators [6,7]. Despite these advantages, modelling the coupled nonlinear response of PGECs under large strains remains challenging, owing to their heterogeneous microstructure and a combination of piezoresistive and interfacial polarization mechanisms. Continuum electro-hyperelastic theories, which combine hyperelastic strain-energy formulations with electrostatic contributions, have successfully described dielectric elastomer actuators (DEAs) and related devices [8,9]. However, their adaptation to graphene-filled systems is limited, with most studies focusing on linear piezoelectricity or decoupled simulations.

In the existing literature, soft nanocomposites are frequently analysed by considering their mechanical and electrical responses separately, even though large deformations and interfacial effects inherently give rise to strong electromechanical coupling. A coupled modelling framework, grounded in continuum theory and finite element implementation, is required to accurately describe how mechanical deformation and electric fields interact and jointly govern the macroscopic behaviour of soft nanocomposites. The present work addresses this gap by formulating an electromechanical coupling model for PGECs under uniaxial compression, integrating: (i) a two-parameter Mooney–Rivlin strain energy density for large-deformation mechanics; (ii) Helmholtz free energy to capture electric field effects; and (iii) finite element analysis in COMSOL Multiphysics^®^ v5.5 [10] to solve the coupled boundary value problem. Experimental measurements of dielectric permittivity, mechanical response, and apparent piezoelectric coefficients are used to calibrate and validate the model. By combining analytical derivations, numerical simulations, and experimental data, this study establishes a predictive framework for the coupled electromechanical behaviour of PDMS–graphene composites.

To the best of our knowledge, no prior study has systematically integrated Mooney–Rivlin hyperelasticity, electromechanical coupling, and finite element implementation for PGECs with direct comparison to experimental benchmarks. Beyond clarifying the role of filler content on effective electromechanical response, this work provides a generalizable methodology for modelling soft nanocomposites that exhibit apparent piezoelectricity through piezoresistive and interfacial polarization mechanisms.

## 2. Materials and Methods

### 2.1. Sample Preparation

Polydimethylsiloxane (PDMS) (Elastosil RT 620 A/B) supplied by Wacker (Wacker Quimica Ibérica S.A., Barcelona, Spain) was used as the polymer matrix. This material is a room-temperature, addition-curing, two-component silicone rubber. Thermally reduced graphene oxide (av-PLAT-70, Avanzare, Navarrete, La Rioja, Spain) was incorporated as a reinforcing nanofiller at loadings of 0.1, 0.5, and 1 wt.%. The PDMS base (Part A) and curing agent (Part B) were mixed at a mass ratio of 10:1. Graphene was first dispersed into Part A using an asymmetric centrifuge, FlackTek SpeedMixer™, model DAC 150.1 FVZ-K (Louisville, CO, USA), at 2000 rpm for 60 s. Subsequently, Part B was added, and the mixture was processed for an additional 60 s under the same conditions. A two-step mixing protocol was adopted to minimise the temperature increase generated during high-speed mixing, as excessive heat can induce premature crosslinking of PDMS and compromise the homogeneity of graphene dispersion. Polymer–graphene elastomer composites (PGECs) were cured at 60 °C at 250 bar, yielding fully crosslinked specimens with controlled geometries.

### 2.2. Experimental Characterization

Mechanical properties were characterised using a universal testing machine, Instron 3366 (Instron, Barcelona, Spain), 50 kN load cell at 23 °C, following ASTM D575-91 [11]. Cylindrical specimens (30 mm diameter, 15 mm height) were subjected to uniaxial compression at a crosshead speed of 5 mm/min up to 40% strain. Stress–strain curves were recorded for control PDMS and PGECs (0.1%, 0.5%, 1% graphene), with nine replicates per composition to ensure reproducibility. Mooney–Rivlin constants (c_10_, c_01_) were derived by fitting stress–stretch data to the two-parameter model.

Dielectric spectroscopy measurements were performed using a Novocontrol ALPHA (Novocontrol, Aachen, Germany) high-resolution dielectric analyser over a frequency range of 10^−1^ to 10^7^ Hz under ambient conditions (23 °C). Square samples with a side length of 10 mm and a thickness of ~1 mm were sandwiched between gold-plated parallel plate electrodes in the dielectric test cell. An alternating voltage of 1 V was applied across the samples during measurement. The frequency-dependent dielectric properties were characterised by determining the complex permittivity [12], ε* = ε′(ω) + jε″(ω), where ε′(ω) represents the real component (dielectric constant) and ε″(ω) represents the imaginary component (dielectric loss factor) as functions of angular frequency (ω). Measurements were averaged over three samples per composition.

The apparent piezoelectric d_33_ coefficient of neat PDMS control samples and PGECs was determined using the Berlincourt method [13] with a PolyK Quasi-Static Piezoelectric meter (PolyK Technologies, State College, PA, USA). The instrument operates within a frequency range of approximately 10 Hz to 1 kHz, with static force controlled via a calibrated knob head in the range of 0.5–5 N (IEEE Std 176-1987) [14]. Films (10 mm in diameter and 1 mm thick) were tested on both surfaces under five load levels. The charge constant d_33_ (pC/N) was obtained as the ratio of generated charge to applied uniaxial force, with negative values indicating polarization opposite to the loading direction. Three replicates per composition were tested to ensure statistical reliability. It should be emphasised that this quasi-static d_33_ value does not necessarily represent an intrinsic piezoelectric effect. Instead, it reflects the effective electromechanical response of the composites, which may arise from a combination of piezoresistive and interfacial polarization mechanisms.

### 2.3. Theoretical Background

To mathematically describe the electromechanical response of PDMS–graphene elastomer composites (PGECs), a continuum electromechanical coupling framework was adopted. The formulation begins with finite-deformation kinematics using the right Cauchy–Green deformation tensor, from which invariants are derived to capture the large-strain response of the elastomer. The internal mechanical behaviour is represented by the two-parameter Mooney–Rivlin hyperelastic model, while electrostatic effects are incorporated through a Helmholtz free energy contribution. The resulting free energy function provides a unified basis for deriving stress measures and electric displacement, which are then implemented both analytically and in a finite element (COMSOL) framework to evaluate the coupled electromechanical performance of the composites under uniaxial compression. Full derivations of the stress and electric field relations are provided in the Supplementary Information.

#### 2.3.1. Kinematic Deformations

For uniaxial compression along the z-axis, the deformation gradient tensor can be written in principal stretches as

(1) $$F=\left[\begin{array}{ccc} \frac{1}{\sqrt{\lambda }} & 0 & 0\\ 0 & \frac{1}{\sqrt{\lambda }} & 0\\ 0 & 0 & \lambda \end{array}\right]$$

where *λ* is the axial stretch in the z-direction. Incompressibility requires that the volume ratio satisfies J = det **F** = 1, leading to equal transverse contractions of *λ*_x_ = *λ*_y_ = *λ*^−1/2^.

From **F**, the right Cauchy–Green deformation tensor is obtained as [15]

(2) $$C=F^{T}F$$

And the two invariants of the deformation tensor, **C**, are expressed as [16]

(3) $$\begin{array}{l} I_{1}=tr(C)\\ I_{2}=\frac{1}{2}\left[\left(tr{\left(C\right)}^{2}-tr(C^{2}\right)\right] \end{array}$$

The Green-Lagrange strain tensor for the hyperelastic structures is defined as

(4) $$\tilde{E}=\frac{1}{2}\left(C-I\right)$$

#### 2.3.2. Hyperelastic Constitutive Model

In this study, the mechanical behaviour of PDMS and PGECs was represented using the two-parameter Mooney–Rivlin strain-energy density function [17,18]. This model is widely regarded as one of the most effective for capturing the large deformation behaviour of elastomers [18,19,20,21].

(5) $$\Psi_{s}(I_{1},I_{2})=c_{10}(I_{1}-3)+c_{01}(I_{2}-3)$$

where *Ψ_s_* is the strain energy density function, and *c*_ij_ are the Mooney–Rivlin constants. These constants were determined by performing nonlinear curve fitting of the experimental stress–strain data using the hyperelastic two-parameter Mooney–Rivlin model module in WELSIM 2025R2 [22] software. This model effectively captures the nonlinear stress–strain response of elastomers under large deformations [17,18,19,20,21]. The two-parameter Mooney–Rivlin model was selected over alternative hyperelastic formulations based on three considerations. First, it provides a reliable description of the nonlinear stress–strain response of silicone elastomers within the strain range studied here (~0–40% compressive strain) using only two parameters, reducing the risk of overfitting inherent to higher-order models. Second, the near-cancellation between c_10_ and c_01_ characteristic of lightly crosslinked PDMS systems (Table 1) is physically meaningful within the Mooney–Rivlin framework, provided the Drucker stability criterion c_10_ + c_01_ > 0 is satisfied, which was confirmed for all compositions. Third, while formulations such as Yeoh and Ogden extend predictive accuracy to wider strain ranges, they require a larger number of material constants and more extensive experimental calibration than the uniaxial compression data available here can reliably support [23].

#### 2.3.3. Electromechanical Coupling

Electromechanical coupling is a widely used experimental and theoretical approach for characterizing dielectric elastomers [24,25,26]. To incorporate the electric field contribution, the total Helmholtz free energy density is defined as [27,28,29,30,31,32].

(6) $$\Psi (I,J,D)=\Psi_{mech}(I,J)+\Psi_{elec}(I,J,D)$$

where **D** is the electric displacement vector and ε = ε0εr is the effective permittivity of the composite, measured experimentally. Under a linear dielectric assumption, **D** = ε**E**, where **E** is the electric field. The constitutive relations follow from the free energy potential [33,34,35,36]

(7) $$\begin{array}{l} P=\frac{\partial \Psi_{mech}}{\partial F}-pF^{-T}\\ \sigma_{c}=\frac{1}{J}PF^{T}\\ S=2\frac{\partial \Psi_{mech}}{\partial C}-pC^{-1}\\ E=\frac{\partial \Psi_{mech}}{\partial D} \end{array}$$

where **F** is the deformation gradient, **P** is the first Piola–Kirchhoff stress, σ_c_ is the Cauchy stress, **S** is the second Piola–Kirchhoff stress, *p* is the hydrostatic pressure enforcing incompressibility, and **I** is the identity tensor. Stress in a material arises from displacement gradients and, in conservative systems, is governed by a single strain energy potential. In hyperelastic dielectric materials, both the material’s elastic deformation and the local electric field influence the stress, while in the absence of an electric field, the standard elasticity equations apply. To capture the electrical effect on the composite, the Maxwell stress should be added to the Cauchy stress to represent electromechanical coupling behaviour, as follows [37]:

(8) $$\sigma_{M}=\left[D⊗E-\frac{1}{2}(D\cdot E)I\right]$$

And for the incompressible case, the total stress, also known as the electromechanical coupling stress, is given by

(9) $$\sigma_{EM}=\frac{1}{J}PF^{T}+\left(D⊗E-\frac{1}{2}(D\cdot E)I\right)$$

For comparison with experimental characterization, the apparent piezoelectric response is discussed in terms of the conventional stress–charge relations [14]:

(10) $$\begin{array}{l} \sigma =c^{E}:S-e^{T}\cdot E\\ D=e:S+\varepsilon^{S}\cdot E \end{array}$$

where **σ** is the stress tensor, **c^E^** is the elastic stiffness tensor at constant electric field, **e** is the piezoelectric stress tensor, **ε^S^** is the permittivity at constant strain, and **S** is the strain tensor. The experimentally measured d_33_ values are interpreted within this framework as effective coefficients arising from interfacial polarization and piezoresistive mechanisms, rather than intrinsic piezoelectricity.

#### Boundary Conditions

In the present study, uniaxial compression was applied along the z-axis, while the lateral surfaces were traction-free, resulting in zero transverse stresses. The electric field was applied across electrodes (E=−∇V) attached to the top and bottom surfaces, with lateral faces electrically insulated.

### 2.4. Numerical Implementation

In this study, COMSOL Multiphysics^®^ v5.5 software was used to support the experimental and theoretical work. COMSOL Multiphysics is widely used to model the electromechanical coupling behavior of piezoelectric composites, allowing for the investigation of phase interactions and mechanisms underlying performance enhancement [38]. The constitutive relations were implemented in COMSOL Multiphysics by coupling the solid mechanics and piezoelectric modules. Then, the electromechanics module was chosen to capture the electromechanical coupling effect in Multiphysics. The experimentally determined permittivity values and Mooney–Rivlin constants were introduced as material parameters, and the electromechanical coupling was defined through the free energy function. This enabled direct comparison between analytical predictions, FEM simulations, and experimental measurements for PGECs under uniaxial compression. The analytical formulation provides an exact solution, whereas the COMSOL finite element approach, being numerical, yields approximate results. Due to the nonlinear nature of hyperelasticity and the additional coupling effects, COMSOL also captures shear strain components not included in the analytical model, which contributes to the systematic differences between the analytical and numerical results discussed quantitatively in Section 3.1.2.

The FEM model geometry consisted of a cylindrical specimen of 30 mm diameter, with thickness h = 8 mm for the purely mechanical validation (Section 3.1.1) and h = 6 mm for the coupled electromechanical simulations (Section 3.1.2). These thicknesses were selected to ensure numerical convergence of the COMSOL solver. In purely hyperelastic simulations at large prescribed displacements, reducing specimen thickness increases the effective compressive strain for a given displacement, intensifying geometric nonlinearity and rendering the tangent stiffness matrix poorly conditioned, which causes Newton iteration convergence difficulties. The introduction of electromechanical coupling adds positive-definite contributions to the tangent stiffness matrix through the dielectric free energy Hessian, improving matrix conditioning and stabilising Newton convergence relative to the purely mechanical case. The thinner geometry (h = 6 mm) was therefore adopted for the coupled analysis, where these regularising contributions are present, while the thicker geometry (h = 8 mm) was used for the uncoupled mechanical validation, where such stabilisation is absent. The domain was discretised using free tetrahedral elements with quadratic (second-order) Lagrange shape functions, as automatically selected by COMSOL’s solid mechanics module for incompressible hyperelastic materials. A standard mesh configuration was adopted, comprising 4069 domain elements, 784 boundary elements, and 92 edge elements, corresponding to 19,687 degrees of freedom (plus one internal DOF); an orthonormal null-space function was applied to ensure numerical stability. Mesh sensitivity was assessed by comparing standard and fine mesh results for the Cauchy stress under pure mechanical loading; the fine mesh yielded values within 2% of the standard mesh, confirming mesh independence. A fully coupled stationary solver was employed with Newton iteration and a relative convergence tolerance of 10^−3^.

## 3. Results and Discussion

**Figure 1:**
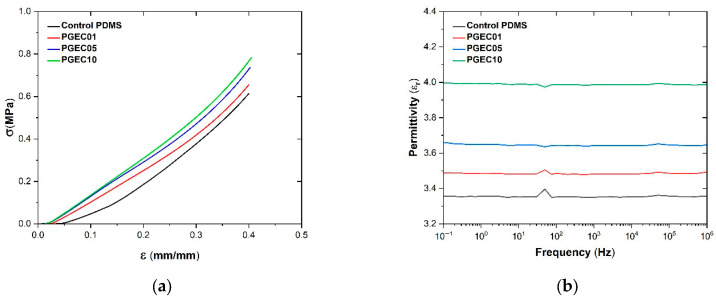
(**a**) Representative stress–strain curves and (**b**) relative permittivity of neat PDMS and PGECs.

PGECs were produced with graphene loadings of up to 1 wt.% to ensure the system remained below the percolation threshold. This preserves the electrically insulating character of the composites, which is essential for electromechanical applications, while enhancing dielectric permittivity [39]. To characterise the effect of graphene loading on the resulting composites, Figure 1a shows the uniaxial compression behaviour of neat PDMS and PGECs. As expected, neat PDMS exhibited a highly compliant response, while the incorporation of graphene resulted in progressive stiffening. This stiffening effect is attributed to the high aspect ratio and strong interfacial interactions of graphene flakes with the PDMS matrix, which restrict chain mobility and distribute stress more effectively across the composite [7]. The stress–strain data were fitted using the two-parameter Mooney–Rivlin model, yielding constants $c_{10}$ and $c_{01}$, and the shear modulus [35], μ = 2($C_{10}$ + $C_{01}$) (Table 1). An increase in c_10_ with graphene loading, coupled with slightly negative c_01_ values, indicates a dominant contribution from the first invariant and a reinforced network structure. Negative c_01_ values are permissible within the Mooney–Rivlin framework, provided the Drucker stability criterion is satisfied. This requires c_10_ + c_01_ > 0, which ensures positive strain energy increments for all deformation modes. The sum of $c_{10}$ and $c_{01}$ remained greater than zero across all compositions, which confirms the stability of the results and prevents unphysical behaviours such as material softening at low strains. This combined value can be regarded as a rough indicator of the initial stiffness of the material, approximating the small-strain shear modulus. This behaviour is consistent with carbon-based filler reinforcement observed in other elastomer systems, where fillers like carbon nanotubes or graphene oxide similarly increase $C_{10}$ while modulating $C_{01}$ to reflect improved cross-linking or entanglement [40]. The monotonic enhancement of stiffness confirms that graphene acts as an effective load-bearing phase while maintaining overall elasticity, making PGECs suitable for applications requiring tunable mechanical properties, such as flexible sensors or actuators [41].

The relative permittivity (ε_r_) measured by broadband dielectric spectroscopy (BDS) increased modestly with graphene content, from 3.4 ± 0.1 for neat PDMS to 4.0 ± 0.2 for PGEC10 (Figure 1b), a ~18% rise at 1 wt%. The enhancement arises from interfacial polarization (Maxwell–Wagner–Sillars effect) and limited charge transport through the sub-percolative graphene network, where graphene flakes create localised dipoles and enhance polarizability without forming a fully conductive pathway [42]. The weak frequency dependence confirms that all composites remain in the insulating regime, consistent with previously reported PDMS–graphene systems [39]. This sub-percolative state is essential for electromechanical performance: exceeding the percolation threshold (typically >1–2 wt% for graphene in PDMS) would shift the composite toward conductive behaviour, increasing dielectric losses and reducing the effective electric field available for electromechanical coupling [3]. The moderate rise in ε_r_ contributes to an increased capacity for electric-field-induced deformation, as higher permittivity amplifies the Maxwell stress contribution in electro-hyperelastic models, thereby enhancing apparent piezoelectric effects. Such dielectric tuning is particularly advantageous for energy harvesting devices, where even modest permittivity gains can improve charge storage and conversion efficiency [43].

Quasi-static d_33_ coefficients measured using the Berlincourt method [13] revealed a clear increase with graphene concentration, from −5.6 ± 0.3 pC/N for pure PDMS to −10.0 ± 0.5 pC/N for PGEC10 (Table 2). The negative sign indicates that the induced polarization is opposite to the applied compressive force. This behaviour is typical of composites dominated by piezoresistive or interfacial polarization mechanisms, rather than intrinsic piezoelectricity. In these systems, charge generation arises from conductivity modulation and asymmetric charge accumulation at filler–matrix interfaces [44]. This apparent piezoelectricity arises because mechanical deformation alters the graphene network’s resistivity, leading to field-dependent charge separation that mimics true piezoelectric response. The corresponding voltage constant g_33_ = d_33_/(ε_0_ε_r_) exhibited a similar trend, confirming enhanced electromechanical sensitivity and dielectric amplification. Although the measured d_33_ values remain below those of ferroelectric polymers such as PVDF (typically −20 to −30 pC/N) [45], the ~80% enhancement observed here demonstrates that conductive fillers can generate significant electromechanical coupling in intrinsically non-polar matrices. This offers a viable pathway toward low-cost, flexible piezocomposites that do not require poling or high-temperature processing [46].

### 3.1. Numerical and Analytical Solutions

#### 3.1.1. Validation Under Pure Mechanical Loading

**Figure 2:**
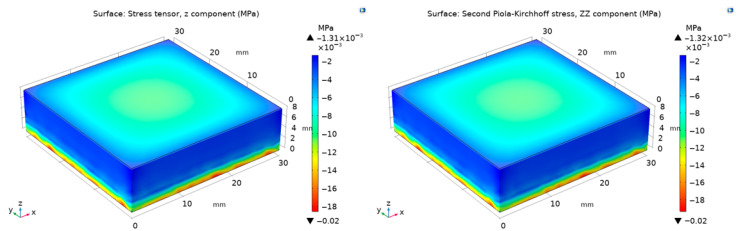
Simulation for PGEC01 of Cauchy (**left**) and second Piola–Kirchhoff (**right**) stresses for the *u*_z_ = −0.1 mm.

Before analysing the electromechanical coupling, the analytical framework was validated under purely mechanical compression to ensure the hyperelastic model’s fidelity in capturing large-deformation behaviour without electrical influences. As a representative example, the PGEC01 composite was subjected to a prescribed axial displacement of u_z_ = −0.1 mm (corresponding to a small compressive strain of approximately 1.25% based on the initial 8 mm sample height), and both the Cauchy stress (which represents the true stress in the deformed configuration) and the second Piola–Kirchhoff stress (a work-conjugate to the Green–Lagrange strain, useful for Lagrangian formulations) were computed analytically from Equation (7) and numerically using COMSOL Multiphysics^®^ v5.5. In this work, the hydrostatic pressure *p* was explicitly determined for both stress tensors, enabling a consistent comparison between analytical and numerical frameworks that highlights the role of hydrostatic enforcement in hyperelastic simulations. As summarised in Figure 2 and Table 3, the analytical and FEM-derived stresses show the same sign and order of magnitude, though a discrepancy of approximately a factor of three is observed (−0.00431 MPa vs. −0.00131 MPa for Cauchy stress, and −0.00442 MPa vs. −0.00132 MPa for the second Piola–Kirchhoff stress). To assess mesh sensitivity, the Cauchy stress and the second Piola–Kirchhoff stress were also computed under a fine mesh configuration, yielding values of −0.00134 MPa and −0.00134 MPa respectively—a difference of less than 2% compared to the standard mesh results. This confirms that the factor-of-three discrepancy between analytical and FEM results is not attributable to mesh density. Inspection of the stress field distributions (Figure 2) reveals that the FEM solution is spatially non-uniform: the stress ranges from approximately −0.00134 MPa at the top surface to −0.0184 MPa near the fixed bottom boundary, reflecting the stress concentration induced by the fixed boundary condition and the barreling tendency of the specimen under compression. The analytical model, by contrast, assumes perfectly homogeneous uniaxial deformation and returns a single volume-representative stress value. The reported COMSOL value corresponds to the least-compressed region of the specimen, which explains the systematic offset relative to the analytical prediction. Furthermore, the near-cancellation between c_10_ (0.980 MPa) and c_01_ (−0.911 MPa) results in an effective shear modulus of only μ = 0.138 MPa, which amplifies sensitivity to any deviation from ideal boundary conditions. An additional contributing factor is that COMSOL computes the full three-dimensional Green–Lagrange strain tensor, which includes shear components arising from geometric nonlinearity and boundary-induced barreling; the analytical Mooney–Rivlin formulation, by contrast, assumes strictly uniaxial kinematics in which all shear components vanish identically, introducing a further systematic offset between the two approaches. Nonetheless, both approaches agree in sign and order of magnitude, confirming that the hyperelastic constitutive model and incompressibility enforcement are correctly implemented.

#### 3.1.2. Electromechanical Coupling and Model Correlation

The electromechanical behaviour was analysed by combining mechanical and Maxwell stress contributions according to Equation (9), where the total stress (coupling stress) incorporates hyperelastic, hydrostatic, and electrostatic terms to fully describe the coupled behaviour under large deformations and applied fields. The Cauchy stress, electric displacement, and piezoelectric stress were evaluated according to Equation (10). Although the PGECs do not exhibit intrinsic piezoelectric behaviour, their apparent piezoelectric response under coupled conditions was examined, and the Maxwell stress-induced coupling response and the piezoelectric effect were treated independently. Experimental permittivity and Mooney–Rivlin constant values were incorporated into the analytical model, while COMSOL simulations coupled the solid-mechanics and electrostatics modules to solve the boundary value problem iteratively by using the Minkowski electromechanics stress tensor. The electrical boundary conditions were defined by setting the bottom surface of the composite as ground and assigning the top surface a floating potential. For the geometric boundary conditions, uniaxial deformation was modelled by fully fixing the bottom surface to prevent any displacement, while the top surface was left free to move under the applied displacement.

In the FEM model, the electromechanical coupling response was obtained by incorporating the mechanical stress contribution alongside the Minkowski electromechanical stress formulation. The implementation in COMSOL additionally required the definition of a piezoelectric material matrix to enable computation of the electric displacement field. These effects were treated independently in both the analytical and FEM analyses, and the results were discussed as summarized in Table 4.

The second-order strain tensor, expressed as the Green–Lagrange strain tensor, was formulated for axial shortenings in the *z*-direction (u_z_) of 0.5, 0.75, 1, and 1.25 mm, representing increasing compression levels up to ~21% strain (based on 6 mm thickness in simulations), which probes the nonlinear regime where hyperelasticity becomes prominent. For the piezoelectric response of the composites, the remaining *d*-coefficients (*d*_31_, *d*_15_) required for the coupling matrix were derived from empirical relationships reported in the literature for polymers [47], as |d_33_| = −2.5|d_31_| and d_15_ ≈ d_31_, with d_31_ and d_15_ taking the opposite sign with d_33_. These relations were adopted as a first-order modelling closure in the absence of full experimental tensor data for PGECs. The complete coupling matrix was subsequently constructed to enable full tensorial representation in both analytical derivations and FEM. The Maxwell stress tensor (**σ_M_**), electromechanical coupling stress tensor (**σ_EM_**), Cauchy stress tensor (**σ**), and the electric displacement (**D**), were analytically determined for each composite type, providing closed-form insights into how graphene modulates the response. To better capture the piezoelectric contribution, results were also represented in terms of the piezoelectric stress tensor (**β**), which quantifies the stress generated by electric fields or vice versa. The analytical findings were validated through numerical simulations in COMSOL Multiphysics for all composite types and displacement conditions, allowing for assessment of geometric nonlinearities, field nonuniformities, and potential edge effects not captured in the analytical uniaxial assumption.

Table 4 compares analytical and numerical results for Maxwell stress (**σ**_M_), electromechanical coupling stress (**σ**_EM_), Cauchy stress (**σ**), piezoelectric stress (**β**), and electric displacement (**D**) under four prescribed displacements (−0.5 mm ≤ u_z_ ≤ −1.25 mm) across four composite compositions. For PGEC10, the analytical model predicts ~125% higher **D** and a fourfold increase in **σ**_M_ and **β** compared with neat PDMS at u_z_ = −0.5 mm, underscoring the strong electromechanical enhancement induced by graphene through amplified interfacial polarization and piezoresistive effects.

To quantitatively assess the reliability of the analytical framework, percentage differences between analytical and FEM results were evaluated across all displacement levels and compositions. For Cauchy stress, deviations range from less than 10% at u_z_ = −0.5 mm to approximately 35% at u_z_ = −1.25 mm. Notably, agreement improves with increasing graphene content at moderate strains: at u_z_ = −1.0 mm, Cauchy stress deviations are approximately 15% for PGEC05 and PGEC10, compared to 33–38% for neat PDMS and PGEC01. This trend is consistent with the increased stiffness of filled composites reducing barreling and boundary-induced stress non-uniformities, which are more pronounced in the compliant neat PDMS matrix [48]. These systematic offsets are attributed to the three-dimensional shear strain components present in the FEM solution but neglected under the uniaxial analytical assumption, and are therefore an expected consequence of the modelling simplification rather than a fundamental limitation of the framework.

At u_z_ = −1.0 mm (λ ≈ 0.833, ~16.7% compressive strain), deviations for the coupled electromechanical quantities remain moderate: 33% for Cauchy stress, 38.71% for piezoelectric stress, 40% for electromechanical coupling stress, and 34.40% for electric displacement. At u_z_ = −1.25 mm, Maxwell stress diverges sharply (190%), indicating that geometric nonlinearities at this deformation level are no longer adequately captured by the uniaxial analytical assumption. The analytical model is therefore considered quantitatively reliable up to u_z_ = −1.0 mm (~16.7% compressive strain); beyond this threshold, full three-dimensional FEM simulations are recommended for quantitative predictions.

**Figure 3:**
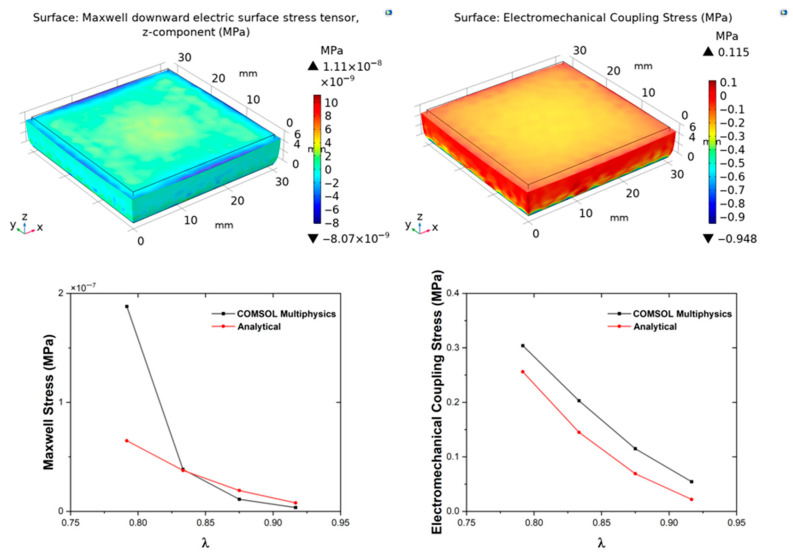
Simulation results for the Control sample. Top panels: COMSOL Multiphysics simulations showing the evolution of Maxwell stress (**left**) and Electromechanical Coupling stress (**right**) as functions of stretch. Bottom panels: corresponding comparative plots between COMSOL and analytical predictions for each parameter, illustrating the agreement between numerical and theoretical results.

**Figure 4:**
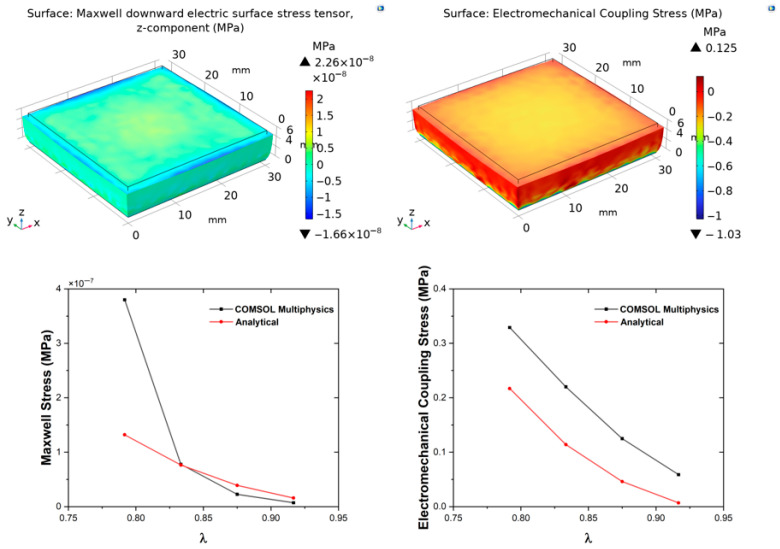
Simulation results for the PGEC01 sample. Top panels: COMSOL Multiphysics simulations showing the evolution of Maxwell stress (**left**) and Electromechanical Coupling stress (**right**) as functions of stretch. Bottom panels: corresponding comparative plots between COMSOL and analytical predictions for each parameter, illustrating the agreement between numerical and theoretical results.

**Figure 5:**
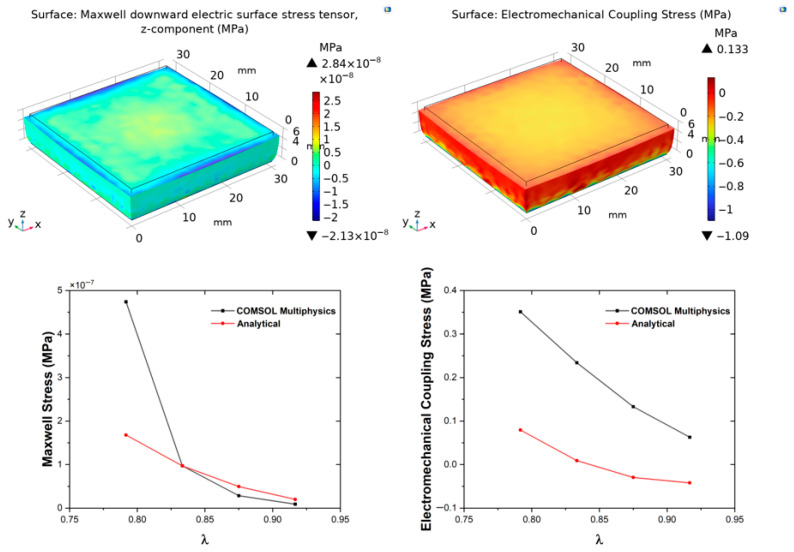
Simulation results for the PGEC05 sample. Top panels: COMSOL Multiphysics simulations showing the evolution of Maxwell stress (**left**) and Electromechanical Coupling stress (**right**) as functions of stretch. Bottom panels: corresponding comparative plots between COMSOL and analytical predictions for each parameter, illustrating the agreement between numerical and theoretical results.

**Figure 6:**
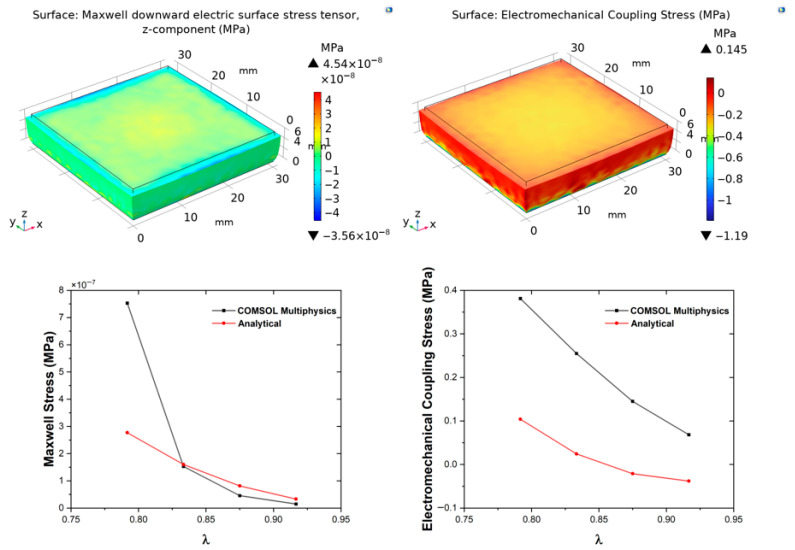
Simulation results for the PGEC10 sample. Top panels: COMSOL Multiphysics simulations showing the evolution of Maxwell stress (**left**) and Electromechanical Coupling stress (**right**) as functions of stretch. Bottom panels: corresponding comparative plots between COMSOL and analytical predictions for each parameter, illustrating the agreement between numerical and theoretical results.

Figure 3, Figure 4, Figure 5 and Figure 6 show the evolution of **σ**_M_ and **σ**_EM_ as functions of stretch *λ* for all composites. The simulation results confirm that deformation remains approximately homogeneous in the central region, with mild stress concentration at the electrode edges (Figure 3, Figure 4, Figure 5 and Figure 6). These localised effects, invisible to the analytical model, become more pronounced at higher filler contents, owing to the increase in stiffness and permittivity. Both analytical and FEM results display monotonic increases with graphene content, confirming that conductive fillers amplify field-induced deformation by enhancing both mechanical reinforcement and dielectric susceptibility, leading to greater Maxwell stress contributions. This filler-dependent amplification is consistent with percolation theory, where even sub-threshold loadings increase effective properties via clustering [49,50,51,52]. The FEM-computed Cauchy stresses show composition- and deformation-dependent agreement with analytical predictions, ranging from less than 10% at low deformation levels to approximately 35% at the highest displacement studied, as discussed quantitatively above.

**Figure 7:**
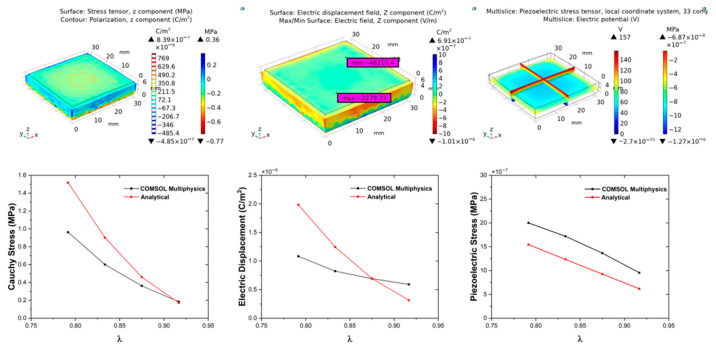
Simulation results for the Control sample. Top panels: COMSOL Multiphysics simulations showing the evolution of Cauchy stress and polarization (**left**), electric displacement and electric field (**middle**), and piezoelectric stress and electric potential (**right**) as functions of stretch. Bottom panels: comparative plots between COMSOL and analytical predictions for each parameter, illustrating the agreement between numerical and theoretical results.

Figure 7 and Table 4 show that the increase in electric displacement (*D*) enhances the material’s energy storage capacity [53]. This provides a crucial parameter for dielectric elastomer actuator and sensor design, enabling the optimization of electromechanical performance and maximizing deformation and sensitivity. All analytical and numerical figures showing the piezoelectric response for other graphene contents are provided in the Supplementary Information (Figures S1–S3). The correspondence between analytical and finite-element results validates the robustness of the proposed electromechanical framework, demonstrating its accuracy in predicting coupled phenomena with an acceptable error margin. The combination of Mooney–Rivlin hyperelasticity with a dielectric free-energy formulation provides a quantitative route to predict the coupled response of elastomers. While previous applications have focused mainly on homogeneous dielectric elastomers, the present work extends this framework to elastomer composites, demonstrating its validity for PDMS–graphene systems [54]. This approach can be further generalized to other electro-hyperelastic materials, such as silicone-based energy harvesters, fiber-reinforced dielectric elastomers, and dielectric elastomer membrane generators [34,55].

The electromechanical enhancement observed across the PGEC series can be directly traced to the sub-percolative microstructural state of the graphene network. The broadband dielectric spectroscopy results (Figure 1b) confirm that all composites remain in the insulating regime, with a weak frequency dependence of ε_r_ consistent with Maxwell–Wagner–Sillars interfacial polarization rather than ohmic conduction. This sub-percolative condition is precisely what enables the observed electromechanical amplification: graphene flakes at loadings of 0.1–1 wt% create localised polarizable regions at the filler–matrix interface without forming a fully conductive pathway, thereby enhancing ε_r_ (from 3.4 to 4.0, ~18%) while preserving the dielectric integrity required for electromechanical coupling. The direct consequence of this permittivity increase is quantified in Table 4, where the Maxwell stress (σ_M_) at u_z_ = −0.5 mm increases from 0.775 × 10^−8^ MPa for neat PDMS to 3.300 × 10^−8^ MPa for PGEC10, representing a fourfold amplification driven predominantly by the ε_r_-squared dependence of the Maxwell stress tensor. Similarly, the electric displacement D at the same deformation level increases from 0.31 × 10^−6^ C/m^2^ to 0.70 × 10^−6^ C/m^2^, consistent with enhanced charge storage capacity at the graphene–PDMS interfaces. The apparent d_33_ coefficients (Table 2) follow the same trend, increasing from −5.6 pC/N to −10.0 pC/N, which is directly attributable to the combined effect of increased permittivity and piezoresistive modulation of the graphene network under compression. Crucially, this enhancement does not require percolation: it is precisely the sub-threshold, interfacially dominated regime that produces a tunable and reproducible electromechanical response. Exceeding the percolation threshold would increase conductivity losses, reduce the effective electric field across the composite, and ultimately degrade rather than enhance the apparent piezoelectric response. These findings therefore establish a clear design principle: maximising interfacial polarization through controlled sub-percolative filler loading is more effective for electromechanical performance than maximising filler content per se.

## 4. Conclusions

The integrated analytical and finite element framework developed in this work establishes a physically grounded methodology for describing the electromechanical behaviour of soft nanocomposites, bridging the gap between material characterisation and device-scale modelling. A key conceptual outcome is that effective piezoelectricity in PGECs is an emergent property governed primarily by relative permittivity, which links mechanical strain, Maxwell stress, electric displacement, and apparent piezoelectric response within a unified formulation. This has direct design implications: by tuning filler content, aspect ratio, and dispersion state, the electromechanical energy conversion efficiency of these composites can be deliberately controlled without relying on intrinsically piezoelectric materials. The validated coupling between the Mooney–Rivlin hyperelastic model and the dielectric Helmholtz free energy formulation provides a transferable tool applicable to other soft nanocomposite systems, supporting the rational design of next-generation flexible sensors, actuators, and energy harvesting devices.

Future work should address the dynamic mechanical characterisation of PGECs, including storage modulus, loss modulus, and damping behaviour, to extend the present quasi-static framework toward frequency-dependent electromechanical response.

## Figures and Tables

**Table 1:** Mooney–Rivlin Constants for PDMS and PGECs.

	Mooney–Rivlin Constants (MPa)		Shear Modulus (MPa)
Graphene Content (%)	c_10_	c_01_	μ
0	0.959	−0.919	0.080
0.1	0.980	−0.911	0.138
0.5	1.005	−0.845	0.320
1.0	1.075	−0.916	0.318

**Table 2:** Piezoelectric constants of PDMS and PDMS–Graphene composites.

Piezoelectric Constants	Materials			
	Control PDMS	PGEC01	PGEC05	PGEC10
d_33_ (pC/N)	−5.6	−7.5	−8.1	−10
g_33_ (Vm/N)	−0.186	−0.242	−0.251	−0.282

**Table 3:** Analytical and numerical stress values obtained for PGEC01 under *u*_z_ = −0.1 mm of displacement.

Stresses (MPa)	Analytical	COMSOL
Cauchy (**σ**)	−0.00431	−0.00131
2nd Piola-Kirchhoff (**S**)	−0.00442	−0.00132

**Table 4:** Comparison of analytical and numerical results for Maxwell stress (*σ***_M_**)_z_, Electromechanical Coupling stress (*σ_EM_*)_z_, Cauchy stress (*σ*)_z_, piezoelectric stress (*β*)_z_, and electric displacement (*D*) obtained under prescribed axial displacements for control PDMS and PGEC composites.

Sample	Quantity	u_z_ = −0.5 mm		u_z_ = −0.75 mm		u_z_ = −1 mm		u_z_ = −1.25 mm	
		Analytical	COMSOL	Analytical	COMSOL	Analytical	COMSOL	Analytical	COMSOL
	**σ_M_** (×10^−8^) (MPa)	0.775	0.349	1.910	1.110	3.750	3.850	6.480	18.80
	**σ_EM_** (MPa)	0.022	0.054	0.069	0.115	0.145	0.203	0.256	0.304
	**σ** (MPa)	0.171	0.184	0.459	0.362	0.902	0.601	1.517	0.963
Control PDMS	**β** (×10^−7^) (MPa)	6.18	9.55	9.26	13.70	12.40	17.20	15.40	20.00
	**D** (×10^−6^) (C/m^2^)	0.31	0.59	0.70	0.69	1.25	0.82	1.98	1.08
	**σ_M_** (×10^−8^) (MPa)	1.580	0.711	3.900	2.260	7.640	7.780	13.20	38.00
	**σ_EM_** (MPa)	0.007	0.059	0.046	0.125	0.114	0.220	0.217	0.329
	**σ** (MPa)	0.186	0.200	0.498	0.392	0.977	0.601	1.644	1.044
PGEC01	**β** (×10^−7^) (MPa)	12.60	19.50	19.00	27.80	25.20	35.10	31.50	40.90
	**D** (×10^−6^) (C/m^2^)	0.45	0.86	1.01	1.01	1.80	1.20	2.88	1.57
	**σ_M_** (×10^−8^) (MPa)	2.000	0.900	4.950	2.840	9.700	9.700	16.80	47.40
	**σ_EM_** (MPa)	−0.042	0.063	−0.029	0.133	0.009	0.234	0.079	0.351
	**σ** (MPa)	0.198	0.246	0.531	0.524	1.042	0.880	1.752	1.140
PGEC05	**β** (×10^−7^) (MPa)	13.90	25.10	20.90	35.50	25.20	45.10	31.50	51.90
	**D** (×10^−6^) (C/m^2^)	0.56	0.99	1.19	1.19	1.88	1.64	3.01	1.80
	**σ_M_** (×10^−8^) (MPa)	3.300	1.470	8.150	4.540	16.00	15.30	27.70	75.30
	**σ_EM_** (MPa)	−0.038	0.068	−0.021	0.145	0.024	0.255	0.104	0.381
	**σ** (MPa)	0.215	0.268	0.577	0.570	1.133	0.957	1.906	1.210
PGEC10	**β** (×10^−7^) (MPa)	26.40	41.00	39.50	58.20	52.70	73.40	65.90	84.90
	**D** (×10^−6^) (C/m^2^)	0.70	1.61	1.55	1.84	2.79	2.23	4.44	2.40

## Data Availability

The original contributions presented in this study are included in the article/Supplementary Material. Further inquiries can be directed to the corresponding authors.

## Supplementary Materials

The following supporting information can be downloaded at: https://www.mdpi.com/article/10.3390/polym18050623/s1, Derivation of equation: Derivation of Cauchy, Maxwell and Total Stress; Figure S1, Figure S2, and Figure S3: Simulation results for the PGEC01, PCG05, and PCG10 composites, respectively.

## Abbreviations

The following abbreviations are used in this manuscript:

Symbol	Description
**β**	Piezoelectric stress tensor
**σ**	Cauchy stress tensor
**σ***_M_*	Maxwell stress tensor
**σ**_EM_	Electromechanical coupling stress tensor
**D**	Electric displacement
**F**	Deformation gradient tensor
**C**	Right Cauchy–Green deformation tensor
**S**	Second Piola–Kirchhoff stress tensor
**P**	First Piola–Kirchhoff stress tensor
**E**	Green–Lagrange strain tensor
**I**	Identity tensor
***E***	Electric field vector
**ε**	Effective permittivity (ε = ε_0_ε_r_)
**ε_0_**	Permittivity of free space
**ε_r_**	Relative permittivity
**λ, u_z_**	Axial stretch ratio and axial displacement
***p***	Hydrostatic pressure
**Ψs**	Strain energy density function
***c*****_10_****, *c*_01_**	Mooney–Rivlin constants
**μ**	Shear modulus
***I*****_1_****, *I*_2_**	First and second invariants of C
***d*****_33_****, *d*_31_, *d*_15_**	Piezoelectric charge coefficients
***g*****_33_**	Piezoelectric voltage coefficient
***J***	Volume ratio (det F)
