# Supplementary material for: Electromechanical Coupling and Piezoelectric Behaviour of (PDMS)–Graphene Elastomer Nanocomposites
Source: Polymers (Basel). 2026 Mar 2;18(5):623. doi: 10.3390/polym18050623 (PMC12986574; doi:10.3390/polym18050623)
Supplement: Supplementary file 1 [file polymers-18-00623-s001.zip › polymers-4133863-supplementary.pdf]

Supplementary Materials

Derivation of Cauchy, Maxwell and Total Stress

1. Material Constants and Symbols

$$C_1, C_2 : \text{Material constants}, \quad \lambda : \text{Axial stretch}, \quad p : \text{Hydrostatic pressure}$$

$$\varepsilon_0 : \text{Vacuum permittivity}, \quad \varepsilon_r : \text{Relative permittivity}$$

$$V : \text{Applied voltage}, \quad d : \text{Thickness}$$

2. Strain Energy Function (Mooney-Rivlin)

$$W(I_1, I_2) = C_1(I_1 - 3) + C_2(I_2 - 3)$$

3. Derivatives of Strain Energy Function

$$W_1 = \frac{\partial W}{\partial I_1} = C_1, \quad W_2 = \frac{\partial W}{\partial I_2} = C_2$$

4. Principal Stretches

$$\lambda_z = \lambda, \quad \lambda_x = \lambda_y = \lambda_\perp = \lambda^{-1/2} \quad \text{(incompressible)}$$

5. Left Cauchy-Green Tensor

$$\mathbf{F} = \begin{bmatrix} \lambda_\perp & 0 & 0 \\ 0 & \lambda_\perp & 0 \\ 0 & 0 & \lambda \end{bmatrix}, \quad \mathbf{B} = \mathbf{F}\mathbf{F}^T = \begin{bmatrix} \lambda_\perp^2 & 0 & 0 \\ 0 & \lambda_\perp^2 & 0 \\ 0 & 0 & \lambda^2 \end{bmatrix}, \quad \mathbf{B}^2 = \begin{bmatrix} \lambda_\perp^4 & 0 & 0 \\ 0 & \lambda_\perp^4 & 0 \\ 0 & 0 & \lambda^4 \end{bmatrix}$$

6. Invariants in terms of λ

$$I_1 = \lambda^2 + 2\lambda_\perp^2 = \lambda^2 + \frac{2}{\lambda}, \quad I_2 = 2\lambda^2\lambda_\perp^2 + \lambda_\perp^4 = 2\lambda + \frac{1}{\lambda^2}$$

7. Cauchy Stress Tensor

$$\sigma_{\text{Cauchy}} = -p\mathbf{I} + 2W_1\mathbf{B} + 2W_2(I_1\mathbf{B} - \mathbf{B}^2)$$

$$\sigma_{\text{Cauchy}} = \begin{bmatrix} \frac{2(C_2\lambda^3 + C_1\lambda + C_2)}{\lambda^2} - p & 0 & 0 \\ 0 & \frac{2(C_2\lambda^3 + C_1\lambda + C_2)}{\lambda^2} - p & 0 \\ 0 & 0 & 2\lambda(2C_2 + C_1\lambda) - p \end{bmatrix}$$

8. Maxwell Stress Tensor

$$\mathbf{I} = \begin{bmatrix} 1 & 0 & 0 \\ 0 & 1 & 0 \\ 0 & 0 & 1 \end{bmatrix}, \quad \mathbf{E} = \frac{V}{d} \begin{bmatrix} 0 \\ 0 \\ 1 \end{bmatrix}, \quad \mathbf{D} = \varepsilon_0 \varepsilon_r \mathbf{E}$$

$$\sigma_{\text{Maxwell}} = \mathbf{D} \otimes \mathbf{E} - \frac{1}{2}(\mathbf{D} \cdot \mathbf{E})\,\mathbf{I}$$

$$\sigma_{\text{Maxwell}} = \begin{bmatrix} -\frac{\varepsilon_0 \varepsilon_r V^2}{2d^2} & 0 & 0 \\ 0 & -\frac{\varepsilon_0 \varepsilon_r V^2}{2d^2} & 0 \\ 0 & 0 & \frac{\varepsilon_0 \varepsilon_r V^2}{2d^2} \end{bmatrix}$$

9. Total Stress Tensor

$$\sigma_{\text{total}} = \sigma_{\text{Cauchy}} + \sigma_{\text{Maxwell}}$$

$$\sigma_{\text{total}} = \begin{bmatrix} \frac{2(C_2\lambda^3 + C_1\lambda + C_2)}{\lambda^2} - p - \frac{\varepsilon_0 \varepsilon_r V^2}{2d^2} & 0 & 0 \\ 0 & \frac{2(C_2\lambda^3 + C_1\lambda + C_2)}{\lambda^2} - p - \frac{\varepsilon_0 \varepsilon_r V^2}{2d^2} & 0 \\ 0 & 0 & 2\lambda(2C_2 + C_1\lambda) - p + \frac{\varepsilon_0 \varepsilon_r V^2}{2d^2} \end{bmatrix}$$

10. Hydrostatic Pressure Solution , $(\sigma_{\text{total}})_{11}=0$

$$p\,(\text{hydrostatic pressure}) = \frac{4C_1 d^2 \lambda + 4C_2 d^2 \lambda^3 + 4C_2 d^2 - \varepsilon_0 \varepsilon_r \lambda^2 V^2}{2d^2\lambda^2}$$

11. Total Stress Component σ_{zz}

$$\sigma_{zz} = \frac{2(\lambda^3 - 1)(C_1\lambda + C_2)}{\lambda^2} + \frac{\varepsilon_0 \varepsilon_r V^2}{d^2}$$

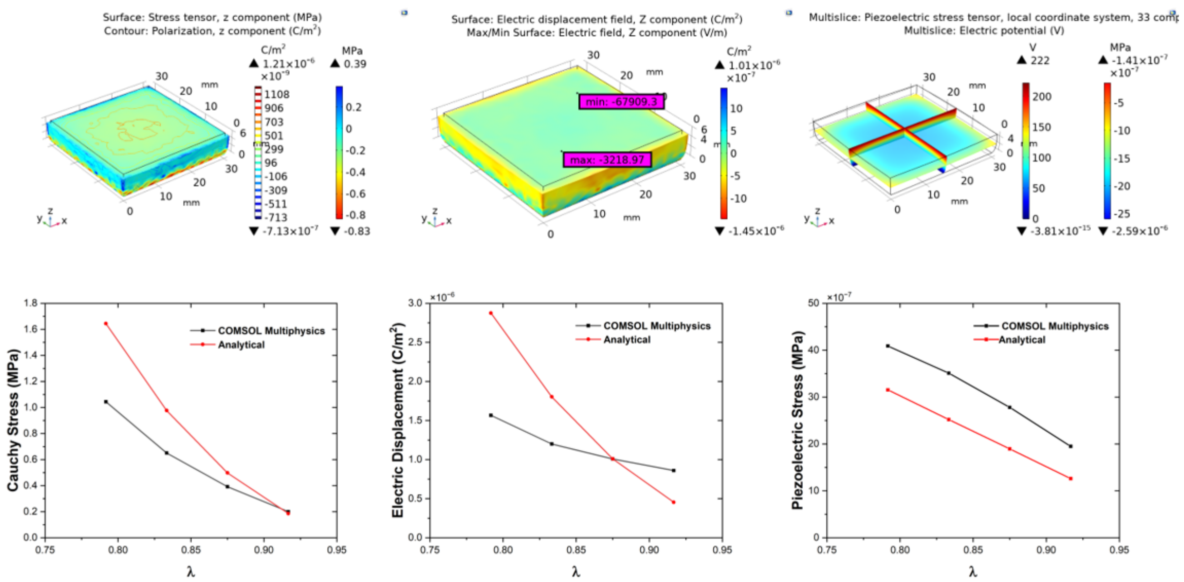

Figure S1. Simulation results for the PGEC01 composite. Top panels: COMSOL Multiphysics simulations showing the evolution of Cauchy stress and polarization (left), electric displacement and electric field (middle), and piezoelectric stress and electric potential (right) as functions of stretch. Bottom panels: corresponding comparative plots between COMSOL and analytical predictions for each parameter, illustrating the agreement between numerical and theoretical results.

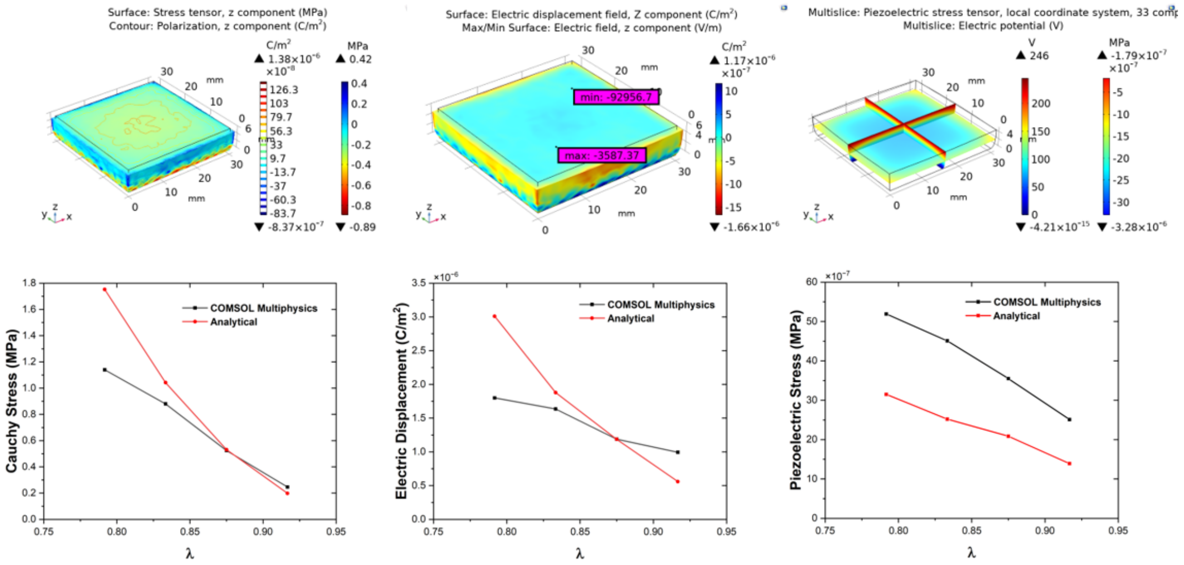

Figure S2 Simulation results for the PGEC05 composite. Top panels: COMSOL Multiphysics simulations showing the evolution of Cauchy stress and polarization (left), electric displacement and electric field (middle), and piezoelectric stress and electric potential (right) as functions of stretch. Bottom

panels: corresponding comparative plots between COMSOL and analytical predictions for each parameter, illustrating the agreement between numerical and theoretical results.

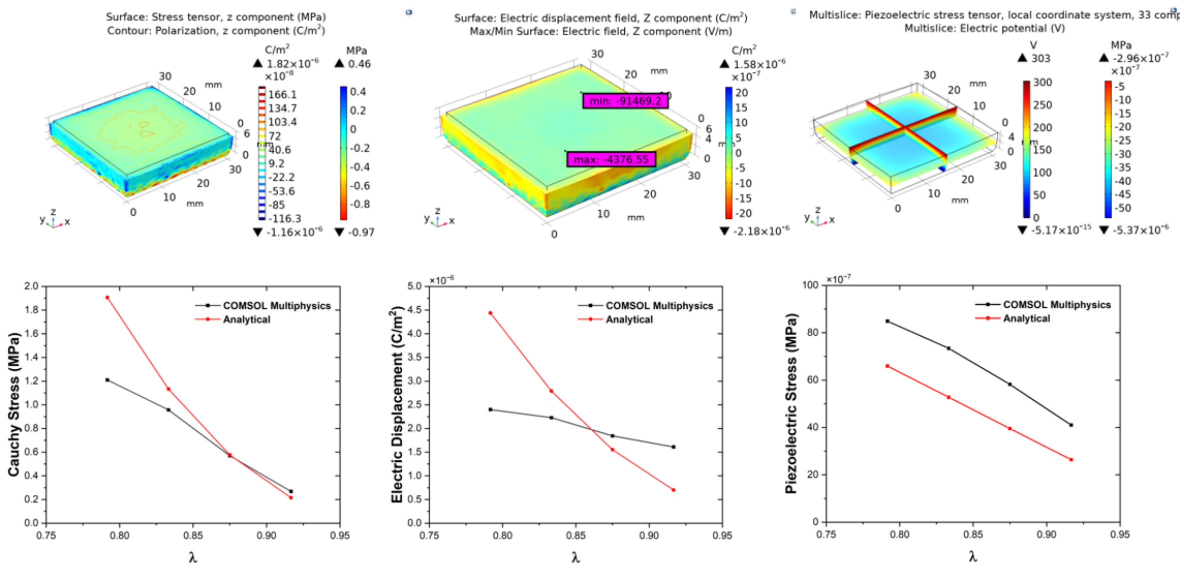

Figure S3. Simulation results for the PGEC10 composite. Top panels: COMSOL Multiphysics simulations showing the evolution of Cauchy stress and polarization (left), electric displacement and electric field (middle), and piezoelectric stress and electric potential (right) as functions of stretch. Bottom panels: corresponding comparative plots between COMSOL and analytical predictions for each parameter, illustrating the agreement between numerical and theoretical results.
